# Acute Effects of Combined and Distinctive Stretching, Foam Rolling, and Eccentric Exercise in Young Men with Hamstring Tightness

**DOI:** 10.5114/jhk/187026

**Published:** 2024-07-17

**Authors:** Sigitas Kamandulis, Phurichaya Werasirirat, Juntip Namsawang, Nutsupa Singhasoot, Audrius Snieckus, Pornpimol Muanjai

**Affiliations:** 1 Institute of Sport Science and Innovations, Lithuanian Sports University, Kaunas, Lithuania.; 2 Exercise and Nutrition Innovation and Sciences Research Unit, Burapha University, Chonburi, Thailand.; 3 Department of Physical Therapy, Allied Health Sciences Faculty, Burapha University, Chonburi, Thailand.

**Keywords:** range of motion, hamstring fascicle length, musculotendinous unit stiffness, muscle oxygenation, strength deficit

## Abstract

This study investigated the changes in fascicle length (FL), musculotendinous unit (MTU) stiffness, muscle oxygen saturation (SmO_2_), and muscular performance following a single bout of a combination of static stretching (SS) or dynamic stretching (DS) with foam rolling (FR), SS only, and eccentric exercise (ECC) only in young men with hamstring tightness. Twenty-five men (20.5 ± 1.5 years) participated in a crossover randomized study of the four conditions (DS+FR, SS+FR, SS, and ECC); each session was spaced seven days apart. FL, MTU stiffness during the straight leg raise (SLR), hamstring SmO_2_, and isometric and eccentric torque were measured before, immediately and 30 min after exercise. Immediately after exercise, the SLR increased significantly by means of 7.4% (d = 1.07), 6% (d = 1.27), 6% (d = 1.10), and 8% (d = 1.04, all p < 0.001) for DS+FR, SS+FR, ECC, and SS, respectively. FL was longer after exercise under all four conditions (p < 0.05). MTU stiffness decreased after ECC (p = 0.038, d = 0.40). SmO_2_ tended to decrease for ECC (p > 0.05), but it was increased immediately after those three exercises. Hamstring isometric torque was significantly reduced by an average of 6–9%, but eccentric torque changes varied among conditions. In conclusion, substantial and similar immediate increases in hamstring flexibility, coupled with reduced isometric torque following various exercises, were accompanied by condition-specific alterations in fascicle length, MTU stiffness, or SmO_2_. These findings provide practical insights for acutely enhancing range of motion in individuals with tight hamstrings.

## Introduction

Hamstring tightness or shortening is the most common musculoskeletal problem in both the general population and athletes (Hamzeh Shalamzari et al., 2022; Erol et al., 2023). This condition can lead to a reduction of the hip and knee angles, which affects the gait pattern (Erkula et al., 2002), increases the risk of injury (Gunn et al., 2019), and can lead to the clinical symptoms of chronic low back pain (Tamartash et al., 2023) or patellar tendinopathy (Morton et al., 2017). Interventions or procedures that prevent hamstring tightness may reduce the risk of these conditions.

Stretching exercise has long been an effective way to increase the range of motion (ROM) and has been incorporated into warm-up routines before exercise and competition (Hsu et al., 2020; López Mariscal et al., 2021). The techniques used to improve ROM include static stretching (SS), dynamic stretching (DS), and proprioceptive neuromuscular facilitation stretching exercises. However, prolonged SS can have a short negative effect on subsequent performance (Behm et al., 2021), although detrimental effects of DS exercises are comparatively rare (Costa et al., 2014). DS is commonly proposed as an effective technique for both improving flexibility and facilitating lower-extremity muscle performance (Behm and Chaouachi, 2011). In addition to stretching exercise, eccentric exercise (ECC) is another method for increasing the ROM and has the additional benefit of strengthening the muscles (Delvaux et al., 2020; Katsura et al., 2019). A recent study revealed that ECC acutely improves ROM to a similar extent as SS and DS in healthy older women (Muanjai et al., 2022). It is thought that these responses reflect sarcomerogenesis coupled with the storage and utilization of elastic energy and/or contribution of the stretch reflex (Cormie et al., 2010; Kilgallon et al., 2007; Lynn and Morgan, 1994; da Silva and Maior 2022).

Foam rolling (FR) has become a popular part of the warm-up before training and to improve flexibility (Konrad et al., 2022). This technique can increase the ROM immediately after a single bout (Wilke et al., 2020), and this outcome may persist for up to 30 min (Kasahara et al., 2022) without a negative effect on jumping or strength performance (Wiewelhove et al., 2019). A recent review by Konrad et al. (2022) acknowledged similar improvement in ROM after FR as a stretching exercise. Moreover, the inclusion of FR within a DS warm-up appears to increase slightly flexibility and to have a larger effect on power output and agility performance (Anderson et al., 2020). Kasahara et al. (2023) reported that the combined use of SS and FR can yield superior increases in knee flexion ROM compared with the combination of DS and FR regardless of the order in which these interventions are applied.

Research has not yet examined the combined effects of stretching and FR, and responses to stretching only or stretching combined with FR or ECC on muscle morphological and mechanical properties are not well established. There is also limited knowledge about the effects of these exercises on muscle oxygen supply, which is linked to aerobic performance, although previous studies have suggested that oxygen availability increases following SS and DS performed during the warm-up (Brodeur et al., 2022). The present study investigated the changes in flexibility, muscle mechanical properties and oxygen saturation (SmO_2_), and muscular strength following a single bout of various interventions, including SS or DS combined with FR, SS alone, or ECC, in young men with hamstring tightness. The hypothesis was that dynamic exercises (DS+FR) would have a smaller impact on flexibility, fascicle length (FL), and musculotendinous unit (MTU) stiffness compared with completely static (SS), partly static (SS+FR), or mechanically demanding (ECC) exercises, but at the same time would result in greater oxygen availability to the muscles and contribute to strength performance.

## Methods

### Participants

Twenty-five healthy and recreationally active young men with hamstring tightness (passive straight leg raise (SLR) ≤ 80°) (Jeong et al., 2022) were recruited. Their age was 20.5 ± 1.5 years and body mass index 20.9 ± 2.6 kg/m^2^. The exclusion criteria were involvement in resistance or stretching exercises, any musculoskeletal pain in the lower limbs or the lower back during the previous six months, neuromuscular or skeletal problems, regular use of muscle relaxants, and use of whey protein or collagen supplements. All procedures were approved by the Research and Innovation Administration Division of the Burapha University Ethics Committee (approval code: IRB1-055/2566; approval date: 18 May 2023) and were conducted according to the guidelines of the contemporary (2013) revision of the Declaration of Helsinki. Before participating in the study, participants gave their written informed consent. The study protocol was registered in the Thai Clinical Trials Registry (TCTR20230825003).

### Design and Procedures

This study had a randomized crossover within-subject design. Each participant performed four interventions in a random order-balanced sequence created using computerized blocks of six: DS+FR, SS+FR, ECC, and SS alone with a washout period of 7 days between bouts. A few days before the pretest measurements, participants attended a laboratory session in which they were familiarized with the measurement apparatus and FR of the left hamstring muscle 3–5 times to learn the cadence, sensation, and targeted location. Participants were asked to continue their present physical activity level throughout the study and to refrain from alcohol, tobacco, and vigorous activity at least 24 h before each testing day.

**Figure 1:**
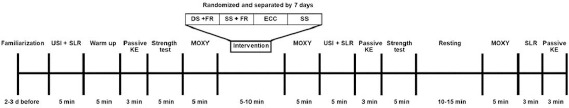
Study timeline before, immediately and 30 min after each intervention. USI, ultrasound imaging; SLR, straight leg raise; KE, knee extension; MOXY, muscle oxygen saturation device; DS+FR, dynamic stretching combined with foam rolling; SS+FR, static stretching combined with foam rolling; ECC, eccentric exercise; SS, static stretching

Before and immediately after exercise, FL, SLR performance, MTU passive stiffness, muscular performance, and SmO_2_ were measured in this order. SLR performance and MTU passive stiffness were measured 30 min after exercise by the same investigators who were blinded to the intervention. The right leg only was investigated in this study. Immediately after the ultrasound imaging and SLR measurements, the participant warmed up with 5 min of unloaded cycling on a cycle ergometer at 60 RPM and then completed the assigned randomized intervention for that day. The study timeframe is presented in Figure 1.

### Measures

#### Hamstring Ultrasound Imaging

Ultrasound imaging B-mode (M5 series, Shenzhen Mindray Bio-Medical, Shenzhen, China) with a linear 4-cm, 7.5-MHz probe (MSK preset) was used to image the right biceps femoris (BF) with the leg straight and the participant in the prone position. The probe was placed 15 cm above the popliteal line, as indicated with a transparent pen marker, and at the same position at the end of SLR testing. FL, muscle thickness (MT) and muscle pennation angle (PA) were analyzed offline using Tracker 6.0.10 software (https://physlets.org/tracker/) with an extrapolated line of the visible fascicle between the superficial and deep aponeurosis (Miyamoto et al., 2020). The average of the two images was used in the analyses.

#### Straight Leg Raise

An inclinometer (ISOMED, Portland, OR, USA) was used to measure passive hamstring extensibility during the SLR test with the participant lying in the supine position and a belt placed over the pelvis to prevent any compensation or counteractive movement. With the inclinometer placed parallel to the distal leg at the medial malleolus, the examiner then passively flexed the participant’s leg upward while keeping the knee straight until resistance or pelvic rotation was detected. This procedure was repeated three times with 15 s of rest in between, and the average of the two closest angles was used in the analysis (Satkunskiene et al., 2020).

#### Knee Flexors Musculotendinous Unit Passive Stiffness

To assess the tissue mechanical properties, a Biodex System Pro 4 isokinetic dynamometer (Biodex Medical Systems, Shirley, NY, USA) was used to measure hamstring MTU passive stiffness. The participant was seated on a chair with the hip flexed at 120° and the shank at 20° below the horizontal (Muanjai et al., 2017). The participant was then asked to relax his leg totally while electromyography of the BF and rectus femoris muscles was recorded. The dynamometer then passively extended the knee at an angular velocity of 5°/s to the maximum end point of discomfort three times. The trial producing the maximum ROM for each movement was noted, and the data for the angle-torque relationship of that trial were selected and analyzed to assess MTU stiffness and peak resistive torque (PRT) at the same maximum ROM for all time points (Muanjai et al., 2022). After gravity correction for the weight of the lower leg, passive stiffness was calculated from the slope of the passive torque-angle curve in the range of 50–80% of maximum ROM using the least-squares method (Cabido et al., 2014).

#### Muscle Strength

Maximal isometric voluntary contraction (MIVC) of the knee flexors and eccentric torque (an index of specific muscle strength) were measured using a Biodex System Pro 4 computerized dynamometer (Biodex Medical Systems) with a sampling rate of 100 Hz. The participant was placed in a position of 85° hip flexion and 50° knee flexion (where 0° = full knee extension) for the MIVC procedure. Before the MIVC, a few submaximal contractions at 50% were performed as a warm-up of the tested muscle, after which the participant rested for 2 min. During the MIVC test, the participant was instructed to inhale, perform knee flexion using maximal effort, and hold this for 3 s. The test was performed twice with a 1-min rest interval between each effort. The maximum MIVC of knee flexor torque was recorded and used in the analysis after gravity correction from the trial producing the maximum torque. After the participant rested for 2 min, eccentric knee flexor torque was tested twice using maximum effort at 60°/s with the knee in the range from 110° of flexion to 10° of extension. The peak eccentric torque and the angle at the peak torque were analyzed Muanjai et al., 2017).

#### Muscle Oxygenation

Near-infrared spectroscopy (Moxy, Fortiori Design LLC, Hutchinson, MN, USA) was used to detect local SmO_2_. The device was placed at the midbelly of the right BF with a nontransparent elastic band wrapped up to the sensor to prevent unexpected external light interrupting the signal. This measurement was taken 5 min before, immediately and 30 min after each exercise bout. The mean values of SmO_2_ over 60-s duration were subsequently examined offline at the end of the initial resting period, immediately following the completion of the intervention protocol, and at the termination of the final 30-min rest period (Brodeur et al., 2022; McManus et al., 2018). The coefficient of variation (CV) was 3.2% and the intraclass correlation (ICC) was 0.774 (0.171–0.943) for SmO_2_.

### Interventions

#### Closed-Chain Dynamic Stretching Plus Foam Rolling (DS+FR)

The DS+FR intervention was based on findings from previous studies (Chen et al., 2018; Kasahara et al., 2023) reporting on the performance of DS as a type of closed kinetic chain exercise. The participant stood on the right leg with the knee flexed 10–15° and bent forward until reaching the end of the hip flexion motion while lifting the contralateral leg toward the back to maintain a straight back. From this point, the participant was instructed to extend the knee slowly until feeling the sensation of maximum discomfort without pain at a cadence of 1 s alternating with 1 s of knee flexion. This procedure was performed for three sets of 60 s each with 15-s rest intervals between sets. To maintain balance during this movement, the participant was allowed to place his hand on the back of the chair or a wall.

After DS exercise, FR was performed on the floor. The roller was placed under the right thigh with the participant in an upright sitting position, the right leg straight, and the left leg across above the right knee. FR started from the popliteal fossa, continued slowly to the ischial tuberosity, and returned to the starting point. The participant was asked to apply as much of his body weight onto the roller as tolerable (Hamzeh Shalamzari et al., 2022; Kasahara et al., 2023; Siebert et al., 2022) for 30 cycles within 60 s to a pace set by a metronome (Kasahara et al., 2023). FR was performed for three sets with 15-s rest intervals between each set.

#### Static Stretching (SS)

The participant performed SS in the supine position using a long towel applied under the right foot with the knee straight and raised the leg to the maximum angle tolerable without pain, and this position was held for 60 s by adjusting the hip angle. This movement was performed three times with 15-s rest intervals in between (Muanjai et al., 2022).

#### Static Stretching Plus Foam Rolling (SS+FR)

SS was performed as described above and then followed by FR.

#### Eccentric Exercise (ECC)

ECC was performed with the participant standing on the right leg. The participant bent the right knee to 10–20° and bent the trunk forward until reaching the end of hip flexion while extending the contralateral leg backward to maintain a straight back (single-leg Romanian deadlift T-drop) (Delvaux et al., 2020). After this movement, the participant returned to the starting position. This procedure was performed for three sets of 15 repetitions within 60 s each with 15-s rest intervals between sets. The participant was allowed to use hand support on the chair’s back or the wall to maintain balance during this exercise.

### Statistical Analysis

The sample size for the present study was calculated using the method of Lee et al. (2018) with an error type 1 of 0.05, statistical power of 0.80, f = 0.4 for a priori sample size calculation for repeated-measures analysis of variance (ANOVA) by G*power software; a minimal sample size of 15 was found to be necessary. Since this study design was a within-subject design that included four conditions, the sample size was increased to N = 25 according to the method of Reiner et al. (2022).

The CV and the ICC for measurement of joint flexibility, ultrasound imaging, and MTU stiffness were calculated as described in a recent study (Muanjai et al., 2022). All data were tested for normality using the Shapiro-Wilk test and are presented as the mean and standard deviation (SD) in the tables and as mean and standard error of the mean (SEM) in the figures. To eliminate time variation when comparing exercise protocols, the absolute difference between two time points was expressed as the mean and the 95% confidence interval. One-way ANOVA was used to compare all baseline measurements and characteristics among conditions. Two-way repeated-measures ANOVA (three times × four interventions) was conducted using the Tukey’s post hoc correction. The effect size was reported as the partial eta squared for repeated measures (magnitude of effect: small = 0.02, moderate = 0.13, large = 0.26) (Bakeman, 2005) and as Cohen’s *d* for pairwise comparisons: small (*d* = 0.2), moderate (*d* = 0.5), and large (*d* = 0.8) (Cohen, 1988). An α level of 0.05 was used to identify significant differences. All statistical analyses were performed using IBM SPSS Statistics for Windows (version 24.0; IBM Corp., Armonk, NY, USA).

## Results

The 25 young men with hamstring tightness completed all four interventions in a one-month period. Their baseline values for BF FL, SLR, MTU passive stiffness, muscular performance, and SmO_2_ did not differ significantly for all measurements (*p* > 0.05) among sessions.

**Figure 2:**
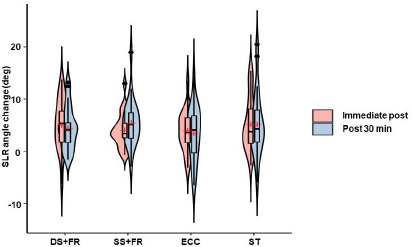
Violin plots illustrating the change distribution frequency of hamstring flexibility from the baseline as measured by the SLR angle (N = 25). Color key: pink, immediately after the intervention; blue, 30 min after the intervention. Embedded boxplots highlight the data distribution for the 25^th^ (bottom) and 75^th^ (top) percentiles. The red dot indicates the median value. DS+FR, dynamic stretching combined with foam rolling; SS+FR, static stretching combined with foam rolling; ECC, eccentric exercise; SS, static stretching

Immediately after all interventions, hamstring flexibility evaluated according to the SLR angle, increased substantially (F_1,96_ = 31.5, *p* < 0.001, η_p_^2^ = 0.25) (Figure 2) by a mean of 7.4% (*d* = 1.07), 6% (*d* = 1.27), 6% (*d* = 1.10), and 8% (*d* = 1.04) (all *p* < 0.001) for DS+FR, SS+FR, ECC, and SS, respectively. At the 30^th^ min, the SLR remained greater than at the baseline by 6.4% (*d* = 1.09), 7.9% (*d* = 1.23), 5.3% (*p* = 0.002, *d* = 0.69), and 8% (*d* = 0.86) (all *p* < 0.001) for DS+FR, SS+FR, ECC, and SS, respectively. There was no interaction effect on the SLR (F_3,96_ = 0.88, *p* = 0.508, η_p_^2^ = 0.03).

The results of muscle ultrasound imaging are shown in Table 1. In the resting position, a notable acute change in BF FL was observed after the intervention (F_1,96_ = 18.6, *p* < 0.001, η_p_^2^ = 0.16) only for DS+FR (*p* = 0.01, *d* = 0.70) and SS (*p* = 0.021, *d* = 0.48). In the extended position during the passive SLR test, longer BF FL was recorded after the intervention (F_1,96_ = 31.5, *p* < 0.001, η_p_^2^ = 0.25). The average increases were 0.73 cm (95% CI: 0.04, 1.49 cm, *p* = 0.029, *d* = 0.39), 0.88 cm (0.11, 1.67 cm, *p* = 0.008, *d* = 0.47), 0.99 cm (0.47, 1.52 cm, *p* = 0.003, *d* = 0.79), and 1.08 cm (0.46, 1.69 cm, *p* = 0.001, *d* = 0.73) for DS+FR, SS+FR, ECC, and SS, respectively. There were no interaction effects of these variables and no time effects on PA and MT.

**Table 1:** Data from BF muscle ultrasound imaging before and immediately after the interventions (N = 25).

Variables	Time points	DS + FR	SS + FR	ECC	SS
**Resting FL (cm)**	Pre	12.03 ± 2.43	11.36 ± 2.12	11.24 ± 2.17	11.68 ± 2.38
	Post	12.51 ± 2.34^*^	11.67 ± 2.52	11.60 ± 1.97	12.12 ± 2.56^*^
**Passive FL (cm)**	Pre	17.55 ± 2.96	18.06 ± 4.17	17.29 ± 3.92	17.67 ± 3.89
	Post	18.28 ± 3.07^*^	18.94 ± 4.55^*^	18.29 ± 4.20^*^	18.74 ± 4.36^*^
**Resting MT (cm)**	Pre	1.75 ± 0.26	1.83 ± 0.34	1.76 ± 0.29	1.84 ± 0.29
	Post	1.80 ± 0.26	1.81 ± 0.31	1.78 ± 0.30	1.82 ± 0.29
**Passive MT (cm)**	Pre	1.81 ± 0.33	1.87 ± 0.35	1.78 ± 0.31	1.79 ± 0.28
	Post	1.85 ± 0.31	1.90 ± 0.32	1.78 ± 0.29	1.81 ± 0.29
**Resting PA (°)**	Pre	7.4 ± 1.7	8.3 ± 2.6	8.0 ± 2.0	8.4 ± 2.9
	Post	7.2 ± 1.9	7.8 ± 2.3	7.5 ± 1.8	7.7 ± 2.2
**Passive PA (°)**	Pre	3.3 ± 1.6	3.8 ± 1.7	3.7 ± 1.7	3.7 ± 1.6
	Post	3.4 ± 1.6	3.7 ± 1.7	3.3 ± 1.7	3.2 ± 1.4

Measurements were obtained before (Pre) and immediately after (Post) the interventions at rest (Resting) and during passive SLR test (Passive). DS+FR, dynamic stretching combined with foam rolling; SS+FR, static stretching combined with foam rolling; ECC, eccentric exercise; SS, static stretching; FL, fascicle length; MT, muscle thickness; PA, pennation angle. Data are expressed as mean and SD. * Significant change between Pre and Post (p < 0.05). No significant group or interaction effects were observed for this variable.

**Figure 3:**
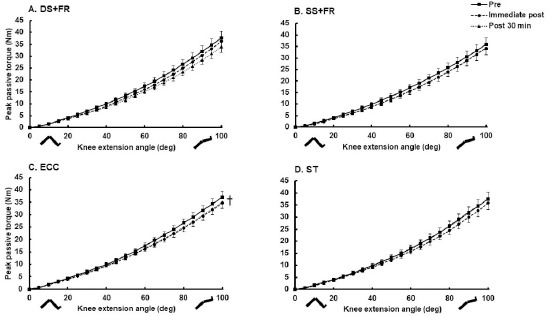
Passive torque-angle changes after the exercise interventions. A. DS+FR. B. SS+FR. C. ECC. D. ST. Data are expressed as the mean and SEM (N = 25). ^†^ Significant change in MTU stiffness and peak passive resistive torque compared from before to immediately after the intervention (p < 0.05)

The knee flexor MTU passive properties are shown in Figure 3. Passive stiffness changed significantly when calculated at the same end points, as indicated by a significant time effect (F_2,188_ = 6.9, *p* = 0.001, η_p_^2^ = 0.07), but there was no interaction effect. Passive stiffness decreased acutely on average by 0.09 Nm/° (0.002, 0.173 Nm/°) (*p* = 0.038, *d* = 0.40) immediately after ECC; only a non-significant trend for an increase in stiffness was observed after the other interventions. However, these values had returned to the pre-intervention level at the 30^th^ min after exercise.

We also assessed the effects of the interventions on muscle performance. There was a significant time effect of the interventions on knee flexor MIVC (F_1,96_ = 89.0, *p* < 0.001, η_p_^2^ = 0.48; Table 2), but no interaction effect. The exercise intervention acutely decreased MIVC on average by 7 Nm (4.7, 9.3 Nm, *d* = 1.25), 6.7 Nm (3.9, 9.4 Nm, *d* = 1.02), 7.1 Nm (3.9, 10.4 Nm, *d* = 0.91), and 5.6 Nm (2.4, 8.8 Nm, *d* = 0.72) (all *p* < 0.001) for DS+FR, SS+FR, ECC, and SS, respectively. A similar time effect was found for peak eccentric torque (F_1,96_ = 14.9, *p* < 0.001, η_p_^2^ = 0.13) (Table 2) as shown by decreases of 8.8% (*p* = 0.011, *d* = 0.55) and 8.8% (*p* = 0.002, *d* = 0.52) after DS+FR and ECC, respectively.

**Table 2:** Data for muscle performance and muscle oxygenation before, immediately and 30 min after the interventions (N = 25).

Variables	Time points	DS + FR	SS + FR	ECC	SS
**Muscle performance**					
**KF MIVC (Nm)**	Pre	80.0 ± 18.9	80.2 ± 19.2	81.1 ± 24.2	79.6 ± 20.2
	Post	73.0 ± 18.7^***^	73.5 ± 16.5^***^	73.9 ± 23.0^***^	74.0 ± 19.2^***^
**KF eccentric torque (Nm)**	Pre	94.2 ± 23.4	91.2 ± 22.5	93.5 ± 27.8	91.0 ± 25.4
	Post	89.7 ± 24.8^*^	89.9 ± 23.1	88.0 ± 24.9^**^	89.0 ± 25.2
**Muscle oxygenation**					
**SmO_2_ (%)**	Pre	75.2 ± 11.0	76.2 ± 8.1	74.7 ± 12.4	76.3 ± 10.2
	Post	80.9 ± 8.4^*^	84.5 ± 7.7^***^	71.6 ± 12.2	82.0 ± 9.7^*^
	Post 30 min	77.3 ± 10.1	81.9 ± 7.7^*^	76.2 ± 11.5	75.8 ± 9.4

Measurements were taken before (Pre), immediately following (Post), and 30 min after the interventions (Post 30 min). DS+FR, dynamic stretching combined with foam rolling; SS+FR, static stretching combined with foam rolling; ECC, eccentric exercise; SS, static stretching; MIVC, maximum isometric voluntary contraction; KF, knee flexors; SmO_2_, muscle oxygen saturation. Data are expressed as the mean and SD. Significant changes between Pre and Post or Post 30 min are indicated as * p < 0.05, ** p < 0.01, and *** p < 0.001. No significant group effect was seen in these variables.

The time effect (F_2,188_ = 9.0, *p* < 0.001, η_p_^2^ = 0.09) (Table 2) and interaction effect (F_6,188_ = 4.5, *p* < 0.001, η_p_^2^ = 0.13) were significant for SmO_2_. Immediately after ECC, BF SmO_2_ tended to decrease non-significantly (*p* > 0.05), but increased significantly by 9.5% (*p* = 0.01, *d* = 0.57), 12% (*p* < 0.001, *d* = 0.89), and 8.8% (*p* = 0.011, *d* = 0.57) for DS+FR, SS+FR, and SS, respectively. SmO_2_ remained higher than the pre-intervention level by an average of 8.8% (*p* = 0.034, *d* = 0.45) at the 30^th^ min after the SS+FR intervention.

## Discussion

Para_Align<thai-distribute>This study’s main findings are the significant and similar improvements in hamstring flexibility after interventions involving stretching combined with FR as either SS alone or ECC. In contrast to our hypothesis, the combination of stretching and FR interfered significantly with subsequent muscle isometric strength performance, possibly because of the acute changes in FL and the trend for reduced MTU stiffness.

The results of the present study are consistent with the findings of a recent meta-analysis and review showing that both stretching and FR interventions increase ROM by 6–8% immediately or 20 min after the interventions (Konrad et al., 2022). It was unexpected that changes in ROM in the current study would be similar among the four interventions, which suggests that they are equally effective for improving short-term flexibility in people with hamstring stiffness.

The mechanism responsible for the improvement in ROM following stretching exercise is explained by a number of factors, such as lesser MTU stiffness, increased FL, proprioceptive changes secondary to changes in the muscle length-tension relationship of the muscle spindle or the Golgi tendon organ, or increased stretch tolerance (Apostolopoulos et al., 2015; Lévenéz et al., 2023; Magnusson et al., 1996). By contrast, FR is thought to increase intramuscular tissue temperature, place direct pressure on the fascia, change tissue hydration, diminish adhesions, and stimulate the Golgi tendon organ, which reduces muscle spindle sensitivity via the Ruffini and Pacinian corpuscles (Hamzeh Shalamzari et al., 2022; Schleip, 2003).

In our study, the DS+FR and SS interventions led to an increase in FL at rest and during passive SLR testing for all interventions, which are likely to reflect changes in viscoelastic stress relaxation or stretch tolerance, as suggested by prior research (Nakamura et al., 2021; Opplert and Babault, 2019). These are a common response following a single session of stretching, FR, or ECC interventions (Morse et al., 2008; Opplert and Babault, 2019). Siebert et al. (2022) also found no impact of FR on the superior ROM gain compared with SS or DS exercise. We speculate that the transverse tension placed on the tissue by FR may be negligible for creating tendon creep compared with a longitudinal force along the MTU generated directly by stretching exercise (Siebert et al., 2022; Wang and Ker, 1995).

The acute improvement in ROM following the ECC bout may be partly attributed to reduced MTU stiffness rather than being influenced solely by changes in FL and stretch perception. A similar trend of reduced MTU stiffness was also observed after other interventions in this study. However, there was no significant reduction in MTU stiffness following SS, possibly because of the short stretching duration of 180 s, given that a recent review has suggested that about 240 s of SS is required to decrease MTU stiffness in both the plantar flexors and hamstrings (Takeuchi et al., 2023). It is also thought that a higher total load of stretching is more effective than a lower load in altering hamstring MTU stiffness (Takeuchi et al., 2021), although this postulation was not confirmed for the SS+FR intervention in this study. Moreover, DS does not seem to lead to an increase in ROM associated with changes in MTU stiffness (Muanjai et al., 2022). By contrast, a brief bout of ECC over a large ROM substantially reduced MTU stiffness, an observation that aligns with previous results obtained after low-intensity eccentric exercise (Nishida et al., 2018; Zhi et al., 2022). Our study contributes to the growing research on the effects of various stretching interventions on MTU stiffness and ROM, although ongoing debate about the mechanisms remains.

It is noteworthy that stretching and FR interventions increased SmO_2_, whereas this effect was not observed during ECC exercise. The increase in SmO_2_ implies that the oxygen supply exceeded the oxygen demand within the muscle (Perrey and Ferrari, 2018), which may be indicative of the favorable facilitation effect of the warm-up. It remains unclear why we found no differences between the types of stretching because a previous study has shown the oxygen saturation level to be higher after DS than after SS (Brodeur et al., 2022). For ECC, there was a noticeable trend toward a decline in SmO_2_, which suggests that, during ECC exercise, the muscle’s oxygen consumption surpasses oxygen delivery (Miranda-Fuentes et al., 2021). Our findings suggest that ECC is effective in changing flexibility, FL, and passive stiffness, but also increases oxygen consumption compared with other warm-up interventions, which render it less suitable for the warm-up before endurance events. Within the context of sports necessitating flexibility, the endorsement of either SS+FR or sole SS for the warm-up may be considered suitable, given the substantial effect size observed in ROM improvement and the absence of a significant effect on eccentric force loss.

The stretch-induced isometric force deficits were remarkable in this study and contradict findings from other studies that reported primarily performance declines following SS lasting over 60 s (Behm et al., 2021). This may be controversial given that DS+FR has been observed to increase or at least preserve athletic performance (Kasahara et al., 2023; Richman et al., 2019). The reasons for the performance deficits remain unclear and may involve a number of neural, morphological, mechanical, or psychological factors, as reviewed in detail by Behm et al. (2021). Our findings suggest that the performance decrease in individuals with hamstring tightness in this study may be related, in part, to acute changes in FL and a trend toward reduced MTU stiffness, which were observed consistently across all interventions regardless of the stretching mode or the inclusion of FR. The investigation conducted by Michalski et al. (2022) similarly revealed a diminution in hamstring muscle force and activity consequent to FR, a phenomenon possibly explicable by a reduction in spinal reflex excitability attributed to a conceivable alteration in excitability of alpha motor neurons, as postulated by Bradbury-Squires et al. (2015). Additionally, we identified intervention-specific differences in the deficits in isometric force and eccentric force, which likely reflected a complex interplay between factors that either facilitate or deteriorate performance. In contrast to prior investigations, Araya-Ibacache et al. (2022) demonstrated an augmentation in the eccentric rate of force development subsequent to DS. It is imperative to acknowledge that the combination of DS with FR might potentially influence performance degradation, warranting further examination.

There are several limitations of this study, which is common in applied science. The absence of clarity about the distinctive FR variables, such as the most effective cadence and FR density, hinders direct comparisons with other studies. The participants’ lack of prior experience with FR, which is more technical than other interventions, may have influenced the treatment efficacy. Methodological differences in the stretching exercise positioning and volume may also have affected the results. These limitations merit consideration during the interpretation of the study's findings, particularly when extrapolating them to other populations, such as young women or older adults.

## Conclusions

The findings from this study suggest that combining FR with stretching is effective for improving ROM in young men with hamstring tightness, but does not yield additional acute ROM benefits compared with hamstring stretching alone. Conversely, ECC produces a similar acute effect on ROM. None of the interventions employed in this study resulted in immediate enhancements in either isometric or eccentric force performance, but stretching and FR interventions increased SmO_2_. Stretch-induced ROM improvement remained 30 min after the exercise interventions. These findings provide practical insights into the effectiveness of specific interventions to improve ROM acutely and the potential to change tissue oxygen saturation to benefit endurance performance.
